# Preprocedural Lp(a) level and ApoB/ApoA-Ι ratio and the risk for contrast-induced acute kidney injury in patients undergoing emergency PCI

**DOI:** 10.1186/s12944-021-01535-4

**Published:** 2021-10-09

**Authors:** Jun Tao, Wen Dai, Chenglin Ye, Qian Yao, Man Zhou, Yan Li

**Affiliations:** 1 Department of Clinical Laboratory, Renmin Hospital of Wuhan University, Jiefang Road 238, Wuchang district, Wuhan, 430060 Hubei China; 2 Department of Urology, Renmin Hospital of Wuhan University, Wuhan, China

**Keywords:** Lipoprotein(a) level, ApoB/ApoA-Ι ratio, Contrast-induced acute kidney injury

## Abstract

**Background:**

High serum Lipoprotein(a) (Lp(a)) level and Apolipoprotein B/Apolipoprotein AΙ (ApoB/ApoA-Ι) ratio are risk factors for cardiovascular disease and kidney disease and have been found to be correlated with the prevalence and prognosis of various kidney diseases. However, it is not clear whether the serum Lp(a) level and ApoB/ApoA-Ι ratio pre-PCI are correlated with the prevalence of contrast-induced acute kidney injury (CI-AKI).

**Methods:**

A total of 931 participants undergoing emergency PCI from July 2018 to July 2020 were included. According to whether the serum creatinine concentration was higher than the baseline concentration (by ≥25% or ≥ 0.5 mg/dL) 48–72 h after contrast exposure, these participants were divided into a CI-AKI group (*n* = 174) and a non-CI-AKI group (*n* = 757). Serum Lp(a), ApoA-Ι and ApoB concentration were detected in the patients when they were admitted to hospital, and the ApoB/ApoA-Ι ratio was calculated. Logistic regression and restricted cubic spline analyses were used to explore the correlation between the Lp(a) concentration or the ApoB/ApoA-Ι ratio and the risk of CI-AKI.

**Results:**

Among the 931 participants undergoing emergency PCI, 174 (18.69%) participants developed CI-AKI. Compared with the non-CI-AKI group, the Lp(a) level and ApoB/ApoA-Ι ratio pre-PCI in the CI-AKI group were significantly higher (*P* < 0.05). The incidence of CI-AKI was positively associated with the serum Lp(a) level and ApoB/ApoA-Ι ratio pre-PCI in each logistic regression model (*P* < 0.05). After adjusting for all the risk factors included in this study, restricted cubic spline analyses found that the Lp(a) level and the ApoB/ApoA-Ι ratio before PCI, within certain ranges, were positively associated with the prevalence of CI-AKI.

**Conclusion:**

High Lp(a) levels and high ApoB/ApoA-Ι ratios before PCI are potential risk factors for CI-AKI.

## Introduction

Contrast-induced acute kidney injury (CI-AKI) refers to acute renal damage caused by the use of contrast agents during angiography or other medical procedures. As a common and serious complication after percutaneous coronary intervention (PCI) procedure in patients with coronary heart disease (CHD), CI-AKI is a significant but reversible and temporary reason for hospital-acquired renal failure. According to the risk factors a patient has, the age of the patient, the type of radiotherapy, and the vascular access site section, the prevalence of CI-AKI ranges from 1.7 to 37.7% [1–4].

The pathogenesis of CI-AKI is not entirely clear. The nephrotoxicity of contrast media, renal medullary ischaemia and hypoxia, oxidative stress, and inflammation are thought to be correlated with the progression of CI-AKI [5]. Dyslipidaemia was also observed to be related to the prevalence of the disease. Dyslipidaemia contributes to CI-AKI by causing endothelial cell damage and inducing inflammation [6, 7]. Studies showed that elevated levels of low-density lipoprotein cholesterol (LDL-C) and small and dense low-density lipoprotein (sdLDL) were important risk factors for CI-AKI [8, 9].

Lipoprotein(a) (Lp(a)) composed of apolipoprotein (a) and LDL-like particles is a polymer complex in the circulatory system. Apolipoprotein B (ApoB) is one of the main components of LDL-C, and it can promote the phagocytosis of oxidized LDL by macrophages and monocytes, which thereby transform into foam cells and induce atherosclerosis [10]. Apolipoprotein AΙ (ApoA-Ι) exists in high-density lipoprotein cholesterol (HDL-C) and maintains the structure of HDL. It can reversely transport cholesterol into the liver and inhibit the oxidation of LDL-C, and it has the effect of preventing atherosclerosis [11]. High levels of Lp(a) and ApoB and low levels of ApoA-Ι are risk factors for cardiovascular disease as well as kidney disease [12–17]. Studies have found that Lp(a) had a significantly inversely correlation with the glomerular filtration rate (GFR) in patients with primary nephropathy, and ApoB/ApoA-Ι was positively correlated with the incidence of chronic kidney disease (CKD) [16, 18]. Lp(a) and ApoB can be deposited in the glomerular mesangial area and stimulate the synthesis of extracellular matrix, leading to glomerular ischaemia and hypoxia, which are associated with the pathogenesis of CI-AKI. ApoA-Ι can promote the reverse transport of cholesterol and inhibit the deposition of cholesterol in the glomerulus, which may have a protective effect against CI-AKI [5, 19]. However, there are no reports on the relationship between the Lp(a) level and ApoB/ApoA-Ι ratio and CI-AKI. Therefore, this study mainly explored the relationships between the pre-PCI ApoB/ApoA-Ι ratio and Lp(a) level and the onset of CI-AKI.

## Methods

### Participants

There were 1446 patients hospitalized in the Department of Cardiology of Renmin Hospital of Wuhan University from July 2018 to July 2020 and underwent emergency PCI procedures. After 515 patients were excluded, 931 patients were enrolled. The exclusion criteria were patients (1) who suffered from severe liver damage; (2) who suffered from infectious diseases, cardiogenic shock, stroke, valvular heart disease, tumours; (3) who suffered from severe kidney damage (eGFR< 30 mL/min/1.73 m^2^) or had had a kidney transplantation; (4) who had incomplete basic data such as on drug use or postoperative creatinine level data; and (5) who were allergic to artificial contrast agents and had been exposed to contrast agents 7 days before the procedure.

### Clinical assessments

After all subjects were admitted to hospital, their clinical characteristics were recorded, including age, sex, and complications such as hypertension and diabetes, as well as a history of smoking, drinking and drug use.

### Blood sampling and analysis

When the patients were admitted to hospital, venous blood was collected into the procoagulant tube and the EDTA-K2 anticoagulant tube. Serum was separated from the procoagulant tube and stored in the refrigerator at − 80 °C. A Siemens Advia 2400 automatic biochemical analyser (Siemens, Erlangen, Germany) and its supporting reagents were used to determine Lp(a), ApoB, ApoA-Ι, total cholesterol (TC), triglycerides (TG), TP (total protein), ALB (albumin), urea, Cr (creatinine), UA (uric acid), eGFR, Glu (glucose), HDL-C, LDL-C, sdLDL and hs-CRP (high-sensitivity C-reactive protein). Among them, a microparticle-enhanced transmission immunoassay was used to detect the concentration of Lp(a), and a polyethylene glycol (PEG)-enhanced immunoturbidimetric assay was used to determine the level of ApoA-Ι and ApoB. A SysmexCA-7000 system (Sysmex, Kobe, Japan) was used to detect the red blood cell (RBC) count, haematocrit (HCT) as well as haemoglobin (Hb) concentration. We used the CKD-EPI 2009SCr equation to calculate eGFR.

### Percutaneous coronary intervention and drug therapy

PCI includes stent placement in and balloon dilation of infarct-related blood vessels. The operation was performed by the surgeon in accordance with the interventional procedures. Coronary angiography was performed by injection of iopamidol as a contrast agent. All patients took 180 mg ticagrelor or 300 mg clopidogrel and 300 mg aspirin before the operation. CI-AKI was defined as the serum Cr concentration was higher than the baseline concentration (by ≥25% or ≥ 0.5 mg/dL) 48–72 h after contrast exposure. Normal saline (0.9%) was intravenously injected for 12 h after contrast agent exposure.

### Statistical methods

Analyses were done with SPSS 23.0 and R 3.5.2. Age, TP and ALB were the continuous variables that obeyed a normal distribution, so they are described by mean ± standard deviation. ApoB/ApoA-Ι ratio, Lp(a), TC, TG, RBC, Hb, HCT, Glu, urea, Cr, UA, eGFR, LDL-C, HDL-C, sdLDL, hsCRP, contrast volume and contrast exposure time, which did not obey the normal distribution and are expressed as interquartile range. The two-independent-sample t test or the Mann–Whitney U test were used to compare groups on the averages of continuous variables. The chi-square test t was used to compare the percentages of categorical variables between CI-AKI group and non-CI-AKI group. Simple and multiple logistic regression were used to explore the relationship between Lp(a) and ApoB/ApoA-Ι and CI-AKI. Restricted cubic spline analyses were used to explore the nonlinear correlation between the Lp(a) concentration or the ApoB/ApoA-Ι ratio and the prevalence of CI-AKI.

## Results

### Characteristics of participants

Among the 931 participants included, 174 participants developed CI-AKI (18.69%). Compared with the non-CI-AKI group, the CI-AKI group had higher age, urea, baseline Cr, postoperative Cr, UA, hsCRP and Lp(a), ApoB levels as well as ApoB/ApoA-Ι ratio, lower Hb concentration, baseline eGFR and postoperative eGFR, and higher the diuretic medication rate (*P* < 0.05) (Table 1).

**Table 1:** Basic clinical and laboratory characteristics of the two groups

characteristics	CI-AKI group (*n* = 174)	non-CI-AKI group (*n* = 757)	*P* value
Clinical variables			
Age (years)	65.48 ± 11.29	62.21 ± 11.29	0.001
Male(n, %)	129 (74.14)	601 (79.39)	0.129
Smoking (n, %)	48 (27.59)	222 (29.33)	0.648
Drinking (n, %)	22 (12.64)	105 (13.87)	0.671
Hypertension (n, %)	119 (68.39)	474 (62.62)	0.153
Diabetes mellitus(n, %)	48 (27.59)	188 (24.83)	0.452
LVEF< 40% (n, %)	29 (16.67)	100 (13.21)	0.234
Laboratory variables			
Lp(a)(mg/L)	223.50 (110.50, 457.75)	132.00 (71.00, 300.50)	< 0.001
ApoA-Ι(g/L)	1.18 (1.02,1.37)	1.20 (1.08,1.36)	0.394
ApoB(g/L)	0.84 (0.66,1.03)	0.78 (0.63,0.93)	0.002
ApoB/ApoA-Ι ratio	0.71 (0.55, 0.90)	0.64 (0.51,0.78)	0.001
RBC(10^12^/L)	4.45 (3.83, 4.85)	4.47 (4.06, 4.85)	0.275
Hb(g/L)	135.00 (119.25, 147.00)	139.00 (125.00, 150.00)	0.010
HCT(%)	41.36 (39.66,43.91)	42.06 (39.46, 44.86)	0.064
TC (mmol/L)	4.20 (3.21, 4.99)	4.02 (3.30, 4.75)	0.094
TG (mmol/L)	1.43 (0.96, 1.99)	1.32 (0.91, 1.93)	0.220
TP(U/L)	65.18 ± 5.95	64.70 ± 5.94	0.345
ALB(U/L)	39.71 ± 4.30	40.16 ± 3.94	0.179
urea (mmol/L)	6.11 (4.93, 7.50)	5.60 (4.56, 6.74)	0.001
Baseline Cr (μmol/L)	77.50 (65.00, 94.25)	70.00 (60.00, 84.00)	< 0.001
Baseline eGFR (mL/min)	82.68 (64.48, 97.65)	93.60 (81.43, 103.25)	< 0.001
UA (μmol/L)	387.50 (323.50, 487.25)	375.00 (311.50, 447.00)	0.045
Glu (mmol/L)	5.92 (4.94, 7.77)	5.71 (4.82, 7.18)	0.228
HDL-C (mmol/L)	0.93 (0.81, 1.20)	0.98 (0.82, 1.14)	0.712
LDL-C (mmol/L)	2.41 (1.67, 3.18)	2.29 (1.74, 2.96)	0.237
sdLDL (mmol/L)	0.84 (0.60,1.15)	0.79 (0.57,1.11)	0.392
LDL-C/HDL-C ratio	2.41 (1.77,3.32)	2.38 (1.74,3.04)	0.164
hsCRP (mg/L)	5.09 (1.59,18.66)	2.28 (0.45, 8.83)	< 0.001
Postoperative eGFR (mL/min)	67.72 (43.36,81.85)	86.27 (73.53,94.44)	< 0.001
Postoperative Cr (μmol/L)	102.5 (84.75,124.25)	81.00 (69.00,96.50)	< 0.001
Medication(n,%)			
Statin	160 (91.95)	667 (88.11)	0.147
ACEI/ARB	117 (67.24)	523 (69.09)	0.636
β-bloker	140 (80.46)	583 (77.01)	0.325
Calcium channel blocker	16 (9.20)	56 (7.40)	0.423
Diuretics	37 (21.26)	101 (13.34)	0.008
Procedural characteristic			
Contrast volume (mL)	129.00 (120.00,146.25)	130.00 (120.00,142.00)	0.712
Contrast exposure time (min)	56.00 (49.50,65.00)	56.00 (48.00,64.00)	0.374
Multivessel disease(n,%)	77 (44.25)	328 (43.33)	0.825

LVEF, left ventricular ejection fraction; Lp(a), lipoprotein a; ApoA-Ι, apolipoproteins AΙ; ApoB, apolipoproteins B; RBC, red blood cell; Hb, haemoglobin; HCT, haematocrit; TC, total cholesterol; TG, triglycerides; TP, Total protein; ALB, albumin; Cr, creatinine; eGFR, estimated glomerular filtration rate (mL/min/1.73 m2); UA, uric acid; Glu, glucose; HDL-C, high-density lipoprotein cholesterol; LDL-C, low-density lipoprotein cholesterol; sdLDL, small and dense low-density lipoprotein; hsCRP, high-sensitivity C-reactive protein; ACEI/ARB, angiotensin-converting enzyme inhibitor/angiotensin receptor blocker

### Associations between the prevalence of CI-AKI and the ApoB/ApoA-Ι ratio and serum Lp(a) levels

To analyse the association between the prevalence of CI-AKI and the serum Lp(a) level or ApoB/ApoA-Ι ratio, we divided patients into Lp(a) quartiles and calculated the odds ratios (ORs) of their risk of CI-AKI, taking patients in the first Lp(a) quartiles as a reference (Table 2). Similarly, Table 3 describes the ORs and 95% CIs of the prevalence of CI-AKI for ApoB/ApoA-Ι ratio. In the unadjusted Model 1, the ApoB/ApoA-Ι ratio and Lp(a) level were positively correlated with the prevalence of CI-AKI. After age and sex adjusted in Model 2, the results were similar to those of Model 1. After further controlling for RBC, Hb, HCT, TC, TG, TP, ALB, urea, Baseline Cr, UA, Baseline eGFR, Glu, HDL-C, LDL-C, sdLDL, hsCRP, ACEI (angiotensin-converting enzyme inhibitor)/ARB (angiotensin receptor blocker), statins, β-blockers, calcium channel blockers, diuretics, contrast volume, contrast exposure time, multivessel disease, age, sex, smoking, drinking, hypertension, diabetes and LVEF< 40% in Model 3, the association was still statistically significant and changed little. The fully adjusted ORs in Model 3 was 1.907 (95% CI: 1.105–3.292) for those in quartile 4 of serum Lp(a) level (the highest) versus quartile 1 (the lowest) and was 2.607 (95% CI: 1.485–4.580) for those in in quartile 4 of serum ApoB/ApoA-Ι (the highest) versus quartile 1 (the lowest).

**Table 2:** Association of the prevalence of CI-AKI with the serum Lp(a) level before PCI

Lp(a) quartile	n	Conc range, mg/L	OR(95%CI)		
			Model 1	Model 2	Model 3
Quartile 1 (low)	233	< 75	Reference	Reference	Reference
Quartile 2	233	75–143	1.156 (0.682–1.961)	1.120 (0.658–1.908)	1.015 (0.565–1.822)
Quartile 3	233	143–328	1.849 (1.127–3.032)	1.822 (1.108–2.996)	1.849 (1.073–3.184)
Quartile 4 (high)	232	≥328	2.360 (1.456–3.826)	2.241 (1.377–3.645)	1.907 (1.105–3.292)
β			−0.308	− 0.293	− 0.257
SE			0.078	0.078	0.086
*P* for trend			< 0.001	< 0.001	0.003

Model 1 no adjustment

Model 2 adjusted for age, sex

Model 3 adjusted for age, sex, smoking, drinking, hypertension, diabetes, LVEF< 40%, RBC, Hb, HCT, TC, TG, TP, ALB, urea, Baseline Cr, UA, Baseline eGFR, Glu, HDL-C, LDL-C, sdLDL, hsCRP, Statins, ACEI/ARB, β-blockers, calcium channel blockers, diuretics, contrast volume, contrast exposure time and multivessel disease

**Table 3:** Association of the prevalence of CI-AKI with the ApoB/ApoA-Ι ratio before PCI

ApoB/A1 quartile	n	Ratio range	OR(95%CI)		
			Model 1	Model 2	Model 3
Quartile 1 (low)	234	< 0.52	Reference	Reference	Reference
Quartile 2	233	0.53–0.65	1.000 (0.598–1.673)	1.077 (0.641–1.811)	1.191 (0.672–2.113)
Quartile 3	234	0.66–0.79	1.325 (0.810–2.166)	1.342 (0.817–2.203)	1.467 (0.859–2.507)
Quartile 4 (high)	231	≥0.79	2.182 (1.371–3.473)	2.537 (1.577–4.082)	2.607 (1.485–4.580)
β			−0.225	−0.185	−0.281
SE			0.076	0.204	0.093
*P* for trend			0.003	< 0.001	0.002

Model 1 no adjustment

Model 2 adjusted for age, sex

Model 3 adjusted for age, sex, smoking, drinking, hypertension, diabetes, LVEF< 40%, RBC, Hb, HCT, TC, TG, TP, ALB, urea, Baseline Cr, UA, Baseline eGFR, Glu, HDL-C, LDL-C, sdLDL, hsCRP, Statins, ACEI/ARB, β-blockers, calcium channel blockers, diuretics, contrast volume, contrast exposure time and multivessel disease

### Lp(a) level and ApoB/ApoA-Ι ratio before PCI and CI-AKI

**Fig. 1:**
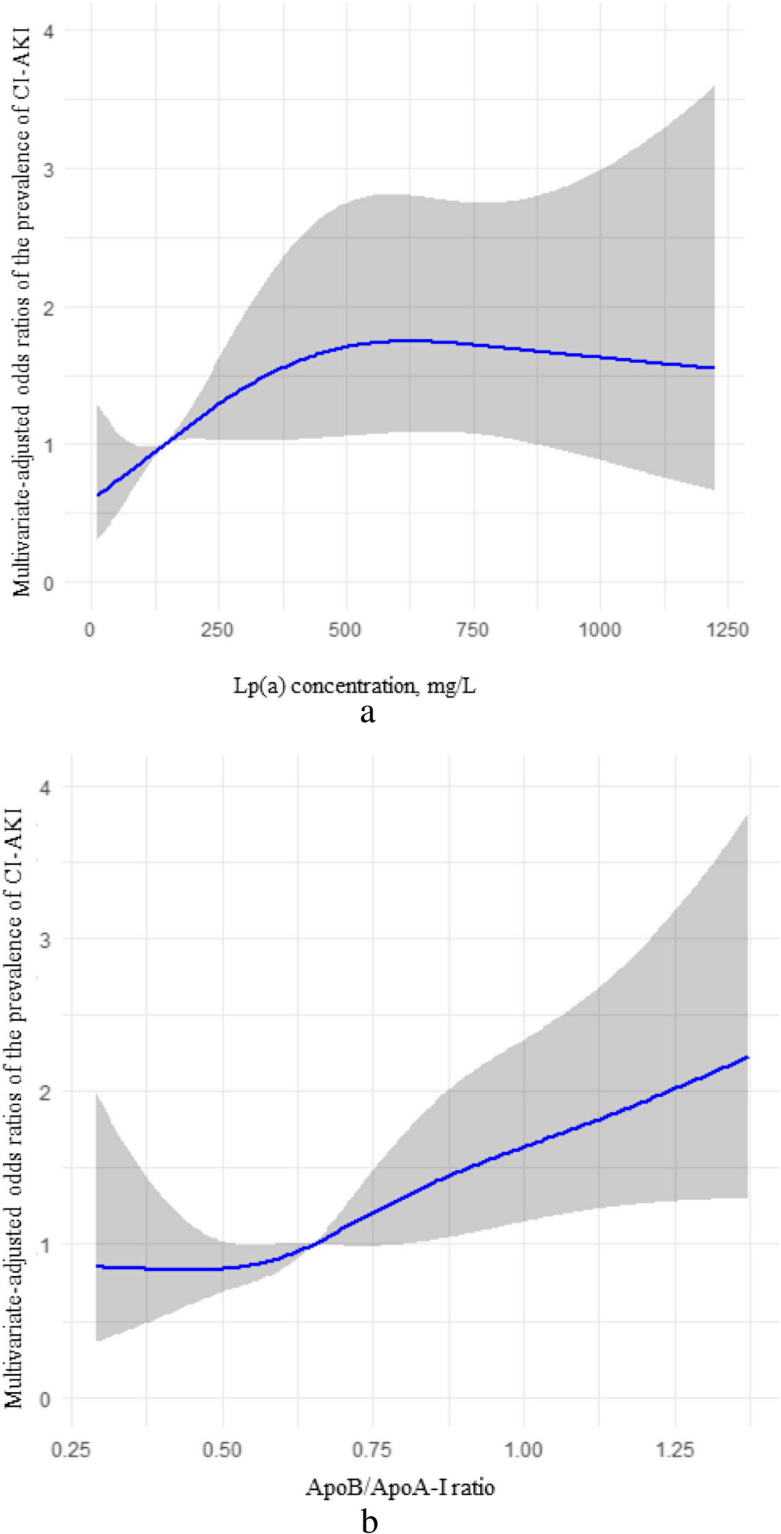
Nonlinear associations between Lp(a) concentration and ApoB/ApoA-I ratio and the prevalence of CI-AKI.

Restricted cubic spline analyses found that after adjusting for all confounding factors included in this study, when the Lp(a) concentration was less than 500 mg/L, the prevalence of CI-AKI increased with Lp(a) concentration. When the Lp(a) concentration was greater than 500 mg/L, the prevalence of CI-AKI reached a plateau. When the ratio of ApoB/ApoA-Ι was less than 0.625, it was a plateau for the prevalence of CI-AKI; when the ratio was greater than 0.625, there was positively correlation between the prevalence of CI-AKI and the ApoB/ApoA-Ι ratio (Fig. 1).

The restricted cubic spline model of the ORs of the prevalence of CI-AKI with serum Lp(a) level (Fig. 1A) and the ApoB/ApoA-Ι ratio (Fig. 1B) was built in patients before PCI. The grey area represents the 95% confidence interval. It was adjusted for RBC, Hb, HCT, TP, ALB, urea, Baseline Cr, UA, Baseline eGFR, Glu, TC, TG, HDL-C, LDL-C, sdLDL, hsCRP, statins, ACEI/ARB, β-blockers, calcium channel blockers, diuretics, contrast volume, contrast exposure time, multivessel disease, age, sex, smoking, drinking, hypertension, diabetes and LVEF< 40%.

## Discussion

In the current research, it was the first time to find that preoperatively higher Lp(a) levels and ApoB/ApoA-Ι ratios in patients undergoing emergency PCI procedures are potential predictors of CI-AKI.

With the growing prevalence of CHD and the advancement of medical technology, the PCI procedure is becoming increasingly popular. CI-AKI, as a common and serious postoperative complication, has received much attention. CI-AKI can increase the risk of renal failure, increase the length of hospitalization and increase the cost of hospitalization [20]. There is no standard effective treatment for CI-AKI; therefore, identifying high-risk patients with CI-AKI and preventing the progression of the disease are still the focus of treatment. For CI-AKI risk assessment, many clinical risk scores have been established, the most classic of which is the Mehron score [21]. However, these risk scores require patients to have a complete medical history, including age, diabetes, anaemia, and other clinical information, which poses certain difficulties for the practice of emergency PCI. Lp(a), ApoA-Ι and ApoB are common and easily available clinical indicators that are correlated with the prevalence of not only CHD but also kidney damage. Although only a few studies have evaluated the association between Lp(a) or ApoB/ApoA-Ι and CI-AKI, other blood lipid indexes, such as LDL-C, HDL-C and sdLDL, have been shown to be correlated with CI-AKI [8, 9, 22]. In addition, the good preventive effect of lipid-lowering drugs against CI-AKI also indirectly clarifies the correlation between blood lipids and CI-AKI [23, 24]. This study found that after adjusting for various confounding factors, Lp(a) and ApoB/ApoA-Ι were still positively correlated with the prevalence of CI-AKI. Therefore, the detection of their levels is of great value for identifying CI-AKI high-risk patients and effectively preventing the progression of the disease.

Although the role of Lp(a) and ApoB/ApoA-Ι in the incidence of CI-AKI is still unclear, multiple important mechanisms might be involved. Lp(a) is an LDL-like protein with an Apo(a) group and its level in serum is mostly determined by the *LPA* gene [25–27]. Clarke et al. found that the mutation of the *LPA* gene was the strongest cardiovascular genetic risk factor among 2100 candidate genes for cardiovascular disease [28]. In addition to cardiovascular disease, Lp(a) is involved in renal injury [29]. Low levels of Lp(a) was observed to stimulate the growth of mesangial cells, while high levels of Lp(a) had antiproliferative or toxic effects [30–33]. In addition, reactive oxygen metabolites (ROMs) in the glomerulus can be activated by Lp(a) induced by the pathway that is sensitive to inhibition of protein kinase C, leading to glomerular damage [34]. These effects may cause kidney damage and lead to the development of CI-AKI.

ApoB/ApoA-Ι was found to be correlated with the onset of kidney disease. A cross-sectional studies found that the increased ApoB levels were correlated with the reduced incidence of CKD, while lower serum ApoA-Ι levels and higher ApoB/ApoA-Ι ratios were correlated with a higher incidence of CKD [16]. Moreover, ApoB/ApoA-Ι has been correlated with the mortality of dialysis patients [35]. The influence of ApoB and ApoA-Ι on the pathogenesis of CI-AKI is not entirely clear, but it may be associated with the deposition of lipids in the glomeruli and the occurrence of inflammation. The uptake of ApoB by glomerular macrophages causes it to be deposited in the mesangial area of ​​the glomerulus, leading to glomerular hypertrophy and increasing transforming growth as well as the level of TGF-β, which in turn cause kidney damage [36–38]. ApoA-Ι can inhibit the oxidation of LDL-C, promote the reverse transport and metabolism of TC, prevent the deposition of cholesterol in the glomerulus and the recruitment of inflammatory cells in the kidney, and inhibit the occurrence of inflammation, which may have a protective effect against CI-AKI [39, 40]. The ApoB/ApoA-Ι ratio can reflect the balance between the risk factors for and protective factors against kidney injury. Compared with ApoA-Ι or ApoB alone, it is considered a better predictor of the occurrence and progression of kidney injury.

Previous studies found that some blood lipid parameters such as LDL-C and sdLDL were correlated with the risk of CI-AKI, which was similar to the results of this study [7, 8]. Few studies have explored the nonlinear correlation between blood lipid indictors and the onset of CI-AKI. Our studies found that the Lp(a) level and the ApoB/ApoA-Ι ratio, within certain ranges, were positively correlated with the prevalence of CI-AKI, which may be valuable for further exploring the correlation between blood lipid levels and CI-AKI.

### Study strength and limitations

This study is the first to find that Lp(a) and ApoB/ApoA-Ι are closely related to CI-AKI. Furthermore, the predictive potential of the Lp(a) level and the ApoB/ApoA-Ι ratio is not related to any persistent CI-AKI clinical or laboratory risk factors, such as age, haemoglobin concentration, and renal function. Therefore, these findings have high clinical practical value for the identification of CI-AKI high-risk patients.

However, some limitations are in this study. First, there are racial and regional differences in the level of Lp(a), but the people included in this study were all from central China. The applicability of the conclusions of this study to other regions and populations needs to be further verified. Second, since the relatively small study size may cause a potential low power of our results, further studies on the large-scale population are necessary. Third, the lack of data on the survival rate and long-term prognosis of patients prevented us evaluating the association between the Lp(a) level and ApoB/ApoA-Ι ratio and the long-term prognosis of the patients. Fourth, although we adjusted for many confounding factors, there were still some confounding factors in our patients, such as access site selection, that were not taken into consideration, which may have impacted the results.

## Conclusion

In short, in patients receiving emergency PCI treatment, high Lp(a) levels and high ApoB/ApoA-Ι ratios before PCI are associated with the increased risk of CI-AKI. Our findings indicate that the Lp(a) concentration and ApoB/ApoA-Ι ratio before PCI may help identify high-risk patients with CI-AKI, thus preventing the occurrence of CI-AKI.

## Data Availability

The datasets generated during and analysed during the current study are not publicly available due to privacy or ethical restrictions but are available from the corresponding author on reasonable request.
